# The Effect of Physical Exercise Pretreatment on Spatial Memory and Learning and Function of Mitochondria in the Brain in Type 2 Diabetic Rats

**DOI:** 10.5812/ijpr-135315

**Published:** 2023-04-10

**Authors:** Homayoon Behmadi, Fatemeh Samiei, Marzieh Noruzi, Zahra Halvaei Khankahdani, Shokoufeh Hassani, Maryam Mehdizadeh, Jalal Pourahmad, Ghorban Taghizadeh, Mohammad Sharifzadeh

**Affiliations:** 1 Department of Toxicology and Pharmacology, Faculty of Pharmacy, Tehran University of Medical Sciences, Tehran, Iran; 2 Department of Toxicology and Pharmacology, Faculty of Pharmacy, Shahid Beheshti University of Medical Sciences, Tehran, Iran; 3 Faculty of Pharmacy, Islamic Azad University of Medical Sciences, Tehran, Iran; 4 Pharmaceutical Sciences Research Center, The Institute of Pharmaceutical Sciences, Tehran University of Medical Sciences, Tehran, Iran; 5 Department of Neurosciences, Faculty of Advanced Technologies in Medicine, Iran University of Medical Sciences, Tehran, Iran; 6 Department of Occupational Therapy, Rehabilitation Research Center, School of Rehabilitation Sciences, Iran University of Medical Sciences, Tehran, Iran

**Keywords:** Exercise, Type 2 Diabetes, Streptozotocin, Spatial Memory, Mitochondria

## Abstract

**Background:**

The prevalence of type 2 diabetes mellitus (T2DM) is increasing worldwide, and this issue is one of the major concerns in the pending years. T2DM causes numerous complications, including cognition, learning, and memory impairments. The positive effect of physical exercise as a popular approach has been shown in many chronic diseases. Further, the improvement effects of exercise on cognition and memory impairment have been noticed.

**Objectives:**

This study examines the possible preventative effects of physical exercise on spatial memory attenuation and brain mitochondrial dysfunction caused by T2DM.

**Methods:**

Male Wistar rats received treadmill exercise (30 min per day, five days per week for two or four weeks). Then, T2DM was induced by a high-fat diet and an injection of streptozotocin (30 mg/kg). Spatial learning and memory were assessed by the Morris water maze test. Further, brain mitochondrial function, including reactive oxygen species (ROS) generation, mitochondrial membrane potential (MMP), mitochondrial swelling, outer membrane damage, cytochrome c release, and ADP/ATP ratio, were measured.

**Results:**

Impaired spatial memory in T2DM rats was observed. Furthermore, brain mitochondrial dysfunction was demonstrated proved by increased ROS generation, MMP collapse, mitochondrial swelling, outer membrane damage, cytochrome c release, and ADP/ATP ratio. Conversely, physical exercise, before diabetes onset, significantly ameliorated spatial memory impairment and brain mitochondrial dysfunction.

**Conclusions:**

This study reveals that physical exercise could prevent diabetes-induced spatial memory impairment. Moreover, it could ameliorate brain mitochondrial dysfunction as one of the possible underlying mechanisms of spatial memory impairment in T2DM.

## 1. Background

Adult-onset diabetes mellitus (T2DM), as a multifactorial, hyperglycemic disease, is an important global health concern with a burden on the economy and public health (1). Statistics from the International Diabetes Federation express that in 2017 451 million people had diabetes, which will increase to 693 million by 2045, and 90 - 95% of them will suffer from T2DM (2). High blood glucose levels in the long-term cause generalized micro and macrovascular damages affecting multiple organs, including nerves, and result in various complications (3). A probable mechanism is that high blood glucose level increases oxidative stress because of glucose autoxidation (4). Diabetes affects the plasticity and morphology of the hippocampus by decreasing the presynaptic proteins, hippocampus atrophy, metabolism changes, and synaptic degeneration (5), which may lead to diminished learning and memory (6). Other mechanisms that may be involved include the effect of diabetes on inflammation and reduced growth factors, especially brain-derived-neurotrophic-factor (BDNF) (6). There is a close association between diabetes and mitochondrial dysfunction. Mitochondrial dysfunction causes diabetes, possibly due to increased insulin resistance due to decreased oxidative phosphorylation (7, 8). On the other hand, diabetes causes mitochondrial dysfunction in all tissues. Hyperglycemia increases reactive oxygen species (ROS) production (7, 9, 10). Over-production of ROS causes mitochondrial dysfunction by increasing protein oxidation (11). The World Health Organization recognized exercise as an effective, inexpensive, and easily available lifestyle intervention to alleviate and prevent chronic diseases (12). Human and animal studies have gathered evidence that regular physical exercise is a good non-medicinal approach to ameliorating cognitive dysfunction, including learning and memory impairments, through several mechanisms (13-15). One of these mechanisms is the muscles' crosstalk with the brain through the myokines release, especially vascular-endothelial-growth factor (VEGF) and (BDNF), which leads to the increased bloodstream to the brain because of angiogenesis and improved cognitive function due to the increased volume of the hippocampus (16-21). The mitochondria of the brain have a significant character in the plasticity of synapses essential for memory and learning because of their role in redox regulation, the concentration of calcium adjustment, and ATP production (22-24). Mitochondrial-dependent mechanisms (e.g., effects on the electron transfer chain (ETC) and reduced ROS production) may also have a role in the positive effects of exercise on cognitive functions (21).

## 2. Objectives

This study surveyed the possible preventive effect of physical exercise on the impairment of spatial memory and the function of the mitochondria in the brain in T2DM rats.

## 3. Methods

### 3.1. Animals

Adult male Wistar rats (200 - 220 g) were obtained from the Faculty of Pharmacy, Tehran University of Medical Sciences. Rats were held in standard polypropylene cages (4 in a cage) in a standard condition of temperature, humidity, light cycle, and free access to water and food.

### 3.2. Experimental Design

**Figure 1.**
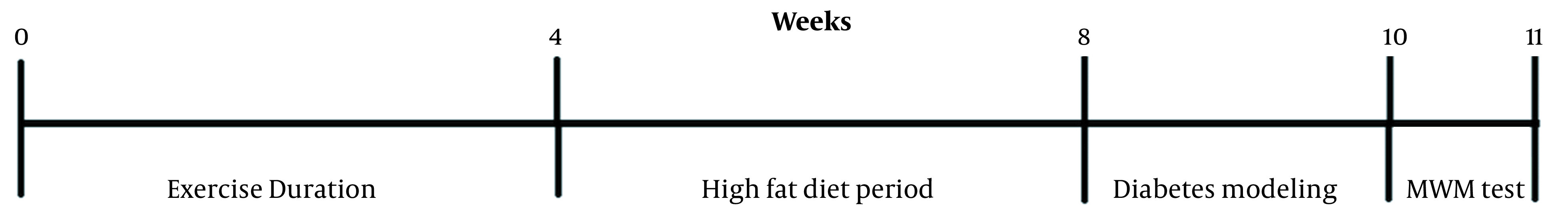
Timeline of study. MWM, Morris water maze

Rats were assigned randomly into six groups (8 in each group) (1) non-diabetic control (rats without any procedure); (2) diabetic control (diabetic rats without any procedure); (3 and 4) non-diabetic groups that received a 2 or 4 week exercise by treadmill; (5 and 6) diabetic groups that received a 2 or 4 weeks exercise by treadmill before induction of type 2 diabetes (Figure 1).

### 3.3. Reagents

Streptozotocin (STZ), ketamine, xylazine, MgCl_2_, 4-2-hydroxyethyl-1-piperazineethanesul folic acid (HEPES), tris-HCl, Na_2_HPO_4_, rotenone, 2′, 7′-dichlorofluorescein diacetate (DCFH-DA), Coomassie blue, sodium succinate, (EGTA), sucrose, KCl, and rhodamine were purchased from Sigma Chemical Co. (St. Louis, MO, USA). Normal and high-fat food (HFD) were purchased from the Royan Institute (Esfahan, Iran).

### 3.4. Induction of Type 2 Diabetes

We used the method described by Zhang et al. (25) and the STZ preparation protocol described by DiaComp protocols (26). Rats were fed a high-fat diet of 45% to 60 % for four weeks. Then, after one night of fasting, rats received STZ (30 mg/kg injected intraperitoneally (IP)). One week after the first injection, rats fasted one night and were again injected with STZ (30 mg/kg). After one week, rats' fasting blood sugar (FBS) was measured through the tail vein. The normal blood glucose level of the rat is 50 - 135 mg/dL. Rats with FBS > 11.1 mmol/L (200 mg/dL) were assumed diabetic (27-29). The diabetic model was stable for at least four weeks. The high-fat diet continued for the duration of the study.

### 3.5. Physical Exercise

Physical exercise was performed using a treadmill. After familiarization of rats with the treadmill running during the first three days (10 min/day 8 m/min speed), the physical exercise treatment started, which involved running for 2 or 4 weeks. The exercise process was similar to all groups as thirty minutes a day for five successive days a week, including two meters per minute for the first five minutes, five meters per minute for the second five minutes, and eight meters per minute for the second five latest twenty minutes. The surface had no slope. The Instrument was equipped with an electrical shocker. Animals were observed during running to monitor the signs of pain or stress (30). At first, the physical exercise periods (2 or 4 weeks) were applied. Then exercise stopped, and the diabetic model was created (10 days), and after that Morris Water Maze (MWM) test was performed (Figure 1).

### 3.6. Spatial Memory and Learning Assessment

Evaluation of spatial memory and learning was performed by MWM. In MWM, the rats find and remember a black-painted circular platform. It is located almost 1 cm under the water in a pool with a 60 cm height and 136 cm diameter (25 ± 2°C). Four 90-second training trials were performed per day for four days. Twenty-four hours after the last training trial, memory retention was evaluated with a probe test. In the probe test, the underwater platform is omitted, and rats are allowed to swim for 90 seconds (31, 32). A camera was placed above the center point of the pool and recorded the behavior of the rats. Fifteen minutes after the probe test, the exposed platform test was performed to check the sensory and motor coordination and the health of the rats' vision, which was similar to the previous days, except that the platform was visible to the rats. Four MWM parameters were recorded, including swimming speed, traveled distance to find the target, and escape latency to find the target in the four first days (training days). Also, in the probe test, the time expended in the target quadrant (Q1) was recorded. Data were analyzed by an Ethovision tracking system (Noldus, the Netherlands).

### 3.7. Evaluation of Mitochondrial Parameters of the Brain

#### 3.7.1. Mitochondrial Isolation

Because previous studies have shown that in addition to the hippocampus, other areas of the brain are also effective in the MWM test (33), the whole brain tissue was isolated. Rats (n = 3) were anesthetized immediately after the probe test by a mixture of xylazine (10 mg/kg) and ketamine (100 mg/kg) IP. All procedure was done on ice to ensure the best preparation. The isolated brain was first homogenized by a glass handheld homogenizer. Then, a two-step task was performed. In the first stage, homogenized tissue was centrifuged at 1500 g for 10 min at 4°C, and the supernatant (which contains mitochondria) was separated. In the second stage, the supernatant was separated at 10000 g for 10 min at 4°C, and mitochondrial sediment was isolated (34). The Bradford method was employed for the protein concentration measurement (35) and was approximately adjusted to 2000 μg/mL. Finally, mitochondrial sediment was prepared in respiration buffer to measure mitochondrial ROS level, mitochondrial membrane potential (MMP) assay buffer for mitochondrial membrane potential assay, swelling buffer for mitochondrial swelling, and tris buffer for mitochondrial cytochrome c release.

#### 3.7.2. The Brain's Mitochondrial ROS Level

Reactive oxygen species level in the brain mitochondrial was determined by a fluorescence spectrophotometry method that used 2′, 7′-dichlorofluorescein diacetate (DCFH-DA) as a fluorescent probe. This substance enters the mitochondria after it is added to the medium and hydrolyzes to dichlorofluorescein. DCFH is non-fluorescent. DCFH then reacts with ROS and forms fluorescent dichlorofluorescein (DCF). Isolated mitochondria were suspended in a respiration assay buffer. After that, DCFH-DA was added to the suspension at a final concentration of 10 μM; then, the suspension was incubated at 37°C for 10 - 15 min. A fluorescence spectrophotometer (Shimadzu RF5000U) measured ROS level at excitation 488 nm and emission 527 nm (36).

#### 3.7.3. The Brain MMP

The uptake of Rh123 by the mitochondria was measured to estimate the brain MMP. Rh123 is a cationic fluorescent dye with a positive charge that goes through the mitochondrial inner membrane with a highly negative (-180 mV) charge. Intact mitochondria uptakes maximum Rh123 because of their inner membrane's highly negative charge. Therefore, the concentration of Rh123 in the environment is the lowest; when the mitochondria are damaged, the charge of its inner membrane absorption of Rh123 reduces, resulting in more Rh123 in the environment. A suspension of isolated mitochondria in the MMP assay buffer was provided. Then, Rh123 was added at the final concentration of 10 µM. The suspension was incubated at 37°C for 10 min. Afterward, the intensity of fluorescence was measured at emission 535 nm and excitation 490 nm by a fluorescence spectrophotometer (Shimadzu RF5000U) (36).

#### 3.7.4. The Brain's Mitochondrial Swelling

To perform this test, an isolated mitochondria pellet was suspended in a swelling assay buffer. This method is based on changes in light scattering measured by a spectrophotometer. An ELISA reader (Tecan, Rainbow Thermo, Austria) measured the absorbance at 540 nm at 30°C. Absorbance decreasing is a sign of increased swelling in the mitochondria (36).

#### 3.7.5. The Brain's Mitochondrial Outer Membrane Damage

We used a cytochrome c oxidase assay kit (Sigma, Germany) according to the manufacturer's protocol. The membrane damage percentage was estimated by comparing the activity of mitochondrial cytochrome c enzyme (ratio) in the absence and presence of a detergent. The measurement is based on colorimetric spectrophotometry. In this test, ferrocytochrome c absorption is measured at a wavelength of 550 nm. Cytochrome c oxidase causes ferrocytochrome c to become ferricytochrome c and reduces its absorption. The greater the damage to the outer membrane of the mitochondria, the greater the amount of cytochrome c oxidase in the environment, and the intensity of absorption decreases.

#### 3.7.6. The Brain Mitochondrial Cytochrome C Release

We used a cytochrome c ELISA kit (Quantikine, UK) as the manufacturer's guideline. This kit is based on a pre-coated monoclonal antibody for cytochrome c into the microplate. Fifty μL of each sample containing one μg of protein was added to wells. This sample was obtained from the supernatant fraction. The microplate was incubated for 2 hours. Following the manufacturer's instructions, wells were measured at 450 nm using a microplate spectrophotometer.

#### 3.7.7. Assay ADP to ATP Ratio

ADP to ATP ratio was measured in whole brain tissue using high-performance liquid chromatography (HPLC) (Waters, USA) with 486 UV-Vis detectors, 510 pumps, and SUPELCOSIL1 LC -18-T (Supelco, Inc., Bellefonte, PA) column. Fresh brain tissue was treated with 6% trichloroacetic acid and centrifuged for 10 minutes at 10000 g. Then, the supernatant was carefully separated. Its pH was adjusted to 7 with KOH. Then the mixture was injected into HPLC. The ratio of ADP to ATP ratio was calculated using the standard curves of ATP and ADP with an HPLC instrument (37).

### 3.8. Statistical Analysis

For each spatial memory and learning parameter (swimming speed, escape latency, and distance traveled), an average value was calculated over the four training days of the MWM. The main effects of diabetes and exercise duration and the interaction effect of diabetes by exercise on the mean value of these parameters were analyzed with a 2 × 3 (diabetes × exercise duration) two-way analysis of variance (ANOVA). The effects of diabetes and exercise on various parameters of the function of mitochondria in the brain (main effects and interaction effects) were analyzed with a 2 × 2 (exercise × diabetes) two-way ANOVA. For multiple comparisons, Tukey's multiple comparison tests were performed. Statistical significance was set at P < 0.05.

## 4. Results

### 4.1. Spatial Learning and Memory

**Figure 2.**
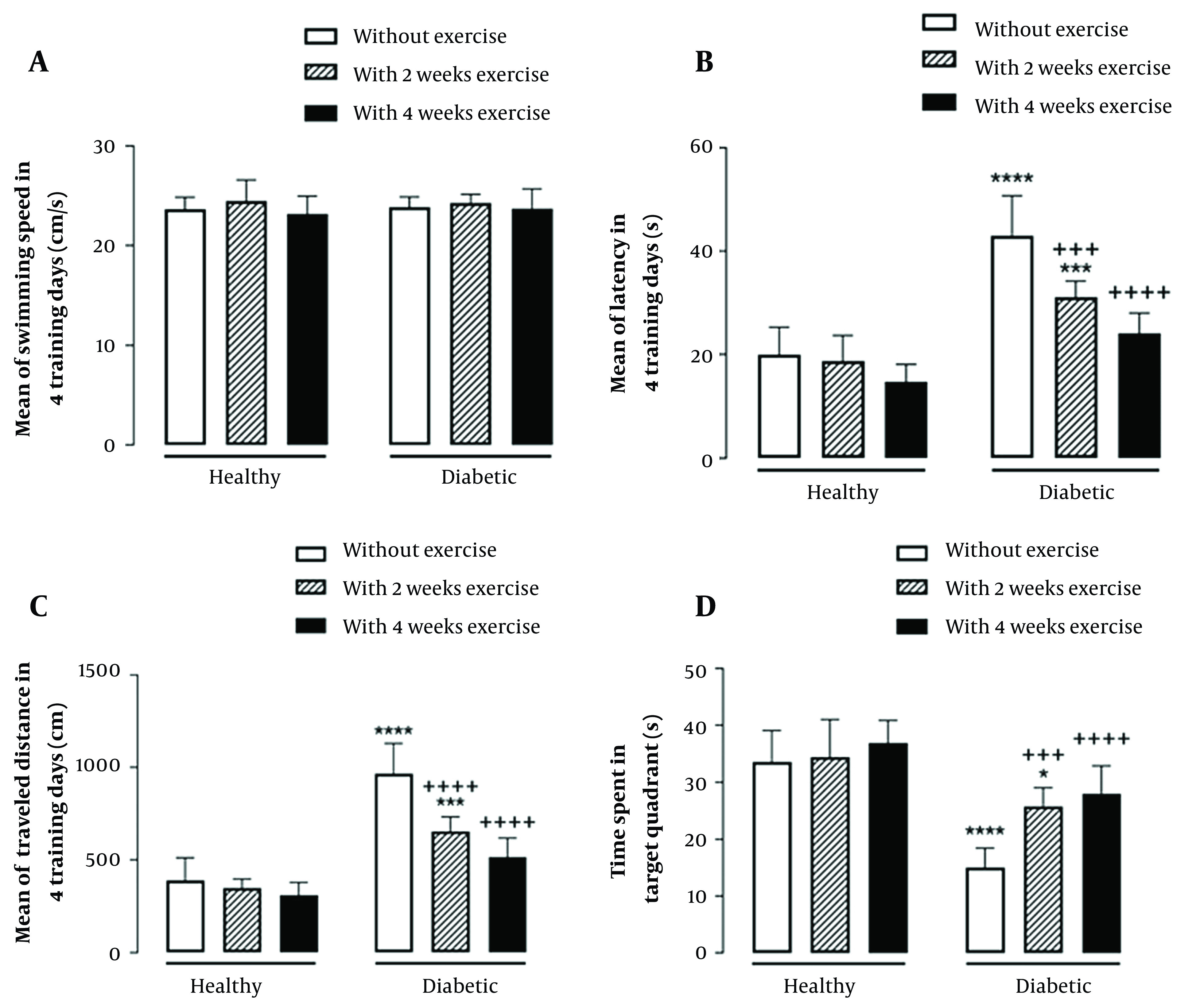
Interaction effect of diabetes by exercise duration on the mean of swimming speed (A), latency (B), and traveled distance (C), in four training days as well as time spent in the target quadrant in the probe test (D) of Morris Water Maze test. * P < 0.05, *** P < 0.001, and **** P < 0.0001 compared with healthy group without exercise; +++ P < 0.001, and ++++ P < 0.0001 compared with diabetic group without exercise.

The results showed that the main effect of diabetes (F _(1, 42)_ = 0.14, P = 0.7) and; exercise duration (F _(2, 42)_ = 1.43, P = 0.25) and the interaction effect of diabetes by exercise duration (F _(2, 42)_ = 0.19, P = 0.83) were not significant for the mean of swimming speed in 4 training days of MWM (Figure 2A).

Furthermore, the main effect of diabetes (F _(1, 42)_ = 107.9, P < 0.0001) and; exercise duration (F _(2, 42)_ = 23.52, P < 0.0001) and their interaction effect (F _(2, 42)_ = 8.28, P = 0.0009) were significant on the mean of escape latency in 4 training days of MWM. The results also indicated the significant main effect of diabetes (F _(1, 42)_ = 142.8, P < 0.0001) and exercise duration (F _(2, 42)_ = 26.21, P < 0.0001), as well as their significant interaction effect (F _(2, 42)_ = 13.41, P < 0.0001) on the mean of traveled distance in 4 training days of MWM. In the probe test of MWM, the main effect of diabetes (F _(1, 42)_ = 75.47, P < 0.0001) and; exercise duration (F _(2, 42)_ =12.3, P < 0.0001) and the interaction effect of diabetes by exercise duration (F _(2, 42)_ = 2.42, P = 0.007) were significant for time spent in the target quadrant. As indicated by multiple comparisons, diabetic rats showed significantly greater escape latency and traveled distance as well as shorter time spent in the target quadrant compared with the control rats. Further, in healthy groups where physical exercise was performed previously to diabetes induction, both 2-week and 4-week exercise resulted in a significant decrease in the escape latency and traveled distance as well as a significant increase in time spent in the target quadrant in diabetic rats. (Figure 2B-D).

### 4.2. Function of Mitochondria in the Brain

#### 4.2.1. Mitochondrial ROS Formation

**Figure 3.**
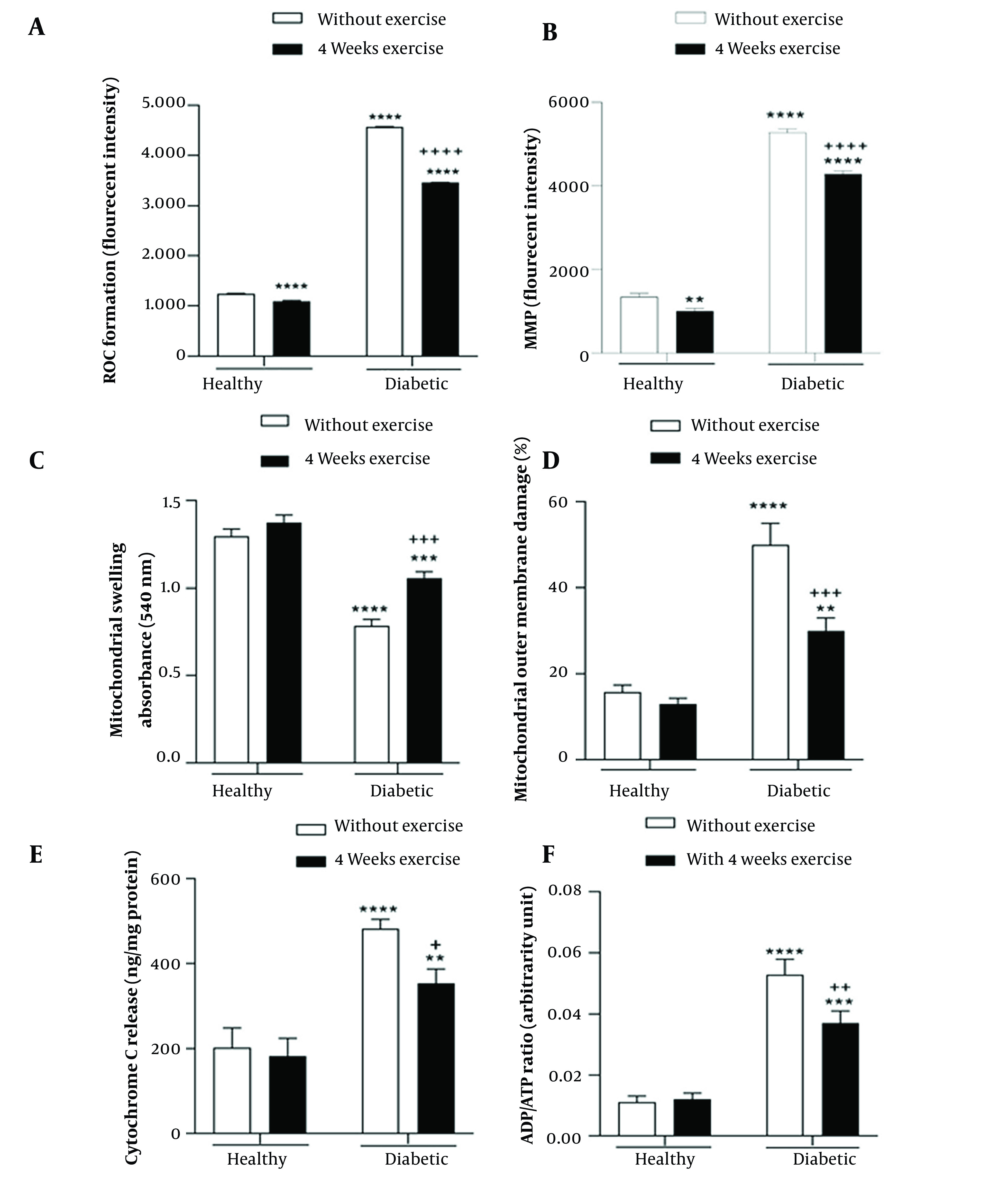
Interaction effect of diabetes by exercise on the reactive oxygen species (ROS) level (A), mitochondrial membrane potential (MMP) (B), mitochondrial swelling (C), mitochondrial outer membrane damage (D), cytochrome c release (E), and ADP to ATP ratio (F) in the brain mitochondria. MMP, mitochondrial membrane potential; ROS, reactive oxygen species. ** P < 0.01, *** P < 0.001, and **** P < 0.0001 compared with healthy group without exercise; + P < 0.05, ++ P < 0.01, +++ P < 0.001, and ++++ P < 0.0001 compared with diabetic group without exercise.

The main effect of diabetes (F _(1, 8)_ = 226542, P < 0.0001) and exercise (F _(1, 8)_ = 10872, P < 0.0001), as well as the interaction effect of diabetes by exercise (F _(1, 8)_ = 6470, P < 0.0001), were significant for brain mitochondrial ROS formation. The results of multiple comparisons demonstrated significantly greater mitochondrial ROS formation in the brain of diabetic rats compared with the control rats. Meanwhile, in groups where physical exercise was performed before diabetes induction, 4-week exercise resulted in a significant decrease in mitochondrial ROS formation in the brain of diabetic rats (Figure 3A).

#### 4.2.2. Mitochondrial Membrane Potential

The brain MMP was significantly affected by the main effect of diabetes (F _(1, 8)_ = 6948, P < 0.0001) and exercise (F _(1, 8)_ = 237.2, P < 0.0001) and their interaction effect (F _(1, 8)_ = 58.23, P < 0.0001). A significant brain MMP collapse was found in the diabetic rats compared with the control rats. Also, results showed that four-week physical exercise attenuated MMP collapse in both diabetic and non-diabetic groups (Figure 3B).

#### 4.2.3. Mitochondrial Swelling

The main effect of diabetes (F _(1, 8)_ = 342.3, P < 0.0001) and exercise (F _(1, 8)_ = 60.82, P < 0.0001), as well as the interaction effect of diabetes by exercise (F _(1, 8)_ = 18.76, P = 0.002), were significant for brain mitochondrial swelling. The results of multiple comparisons indicated that diabetes resulted in brain mitochondrial swelling, while in groups where physical exercise was performed before diabetes induction, 4-week exercise significantly decreased brain mitochondrial swelling in diabetic rats (Figure 3C).

#### 4.2.4. Mitochondrial Outer Membrane Damage

Diabetes (F _(1, 8)_ = 236.1, P < 0.0001), and exercise (F _(1, 8)_ = 75.73, P < 0.0001) main effects, as well as diabetes by exercise interaction effect (F _(1, 8)_ = 54.62, P < 0.0001), were significant for brain mitochondrial outer membrane damage. As depicted in Figure 3D, diabetes caused brain mitochondrial outer membrane damage; however, in groups in which physical exercise was performed before diabetes induction, 4-week exercise significantly decreased brain mitochondrial outer membrane damage in diabetic rats (Figure 3D).

#### 4.2.5. Cytochrome C Release

The cytochrome c release was affected by the main effect of diabetes (F _(1, 8)_ = 110.1, P < 0.0001) and exercise (F _(1, 8)_ = 11.78, P = 0.009), as well as the interaction effect of diabetes by exercise (F _(1, 8)_ = 6.3, P = 0.04). The results of multiple comparisons suggested significantly greater cytochrome c release from brain mitochondrial fraction of diabetic rats compared with the control rats. Conversely, in groups where physical exercise was performed before diabetes induction, 4-week exercise resulted in a significant decrease of cytochrome c release from the brain mitochondrial fraction of diabetic rats (Figure 3E).

#### 4.2.6. Brain ADP to ATP Ratio

The main effect of diabetes (F _(1, 8)_ = 274.8, P < 0.0001) and exercise (F _(1, 8)_ = 13.78, P = 0.006), and the interaction effect of diabetes by exercise (F _(1, 8)_ = 17.69, P = 0.003) were significant for brain mitochondrial ADP to ATP ratio. As shown in Figure 3F, a significantly greater brain mitochondrial ADP to ATP ratio was found in the diabetic rats compared with the control rats. Further, in groups where physical exercise was performed previously to diabetes induction, 4-week exercise led to a significant decrease in brain mitochondrial ADP to ATP ratio in the diabetic rats (Figure 3F).

## 5. Discussion

Type 2 diabetes mellitus, a prevalent chronic disease, is associated with multi-organ dysfunction due to hyperglycemia (2). The most impressive finding of the current study was that physical exercise, when applied before the induction of diabetes, significantly reduced spatial learning and memory impairments, and mitochondrial dysfunction in the brain of diabetic rats. As evidenced by MWM parameters compared to the control group, physical exercise ameliorated spatial memory retention and acquisition (Figure 2A and D). The negative impact of diabetes on memory can be due to several reasons, including decreased neuroplasticity and changes in neuronal metabolism, especially in the hippocampus (5, 6). Another important reason may be the destructive effect of diabetes on the function of the mitochondria, which increases ROS production, which leads to oxidative stress. The effect of mitochondria on the learning process is important because of its contribution to the plasticity of neurons. It supplies large ATP needed by neurons and regulates calcium-dependent signaling and redox regulation (22-24). Our physical exercise protocol and duration periods from 2 to 4 weeks have been used repeatedly in previous studies (38, 39). Also, the findings of our pilot showed that the duration of 2 or 4 weeks could have a significant impression on improving spatial memory parameters as well as mitochondrial parameters (40). Also, studies have shown that exercise at low or moderate intensity is more beneficial than exercise at high intensity in improving spatial memory. The reason is the impression of exercise intensity on the production of BDNF. Also, the increase in ROS production is due to the increase in cellular respiration (13, 15). Therefore, this protocol and duration of physical exercise were chosen. Based on our current result and our previous study (41), as well as the pilot groups of 2, 4, and 6 weeks duration conducted at the beginning of this study, the best duration was observed in the 4-week groups. Therefore, we studied four weeks of exercise for mitochondrial parameters evaluation. Physical exercise also has neuroprotective properties (21). Physical exercise can raise the activity of neurons in the brain, especially in the hippocampus (13, 15, 16), and communicates with different tissues by stimulating the secretion of myokines, including BDNF and VEGF (17). Increasing BDNF can increase cell proliferation and hippocampal neurogenesis (13, 42). Exercise stimulates angiogenesis in the brain by increasing VEGF (21). Physical exercise slows the decrease in hippocampus volume due to aging (19, 43). Exercise also increases synaptic plasticity and neurogenesis, which is significant in the learning and memory process (16, 44). Physical exercise has anti-neuro-inflammatory properties (39). Furthermore, reducing BAD and Bax (as apoptosis markers) and increasing BcL2 (an anti-apoptosis marker) can reduce apoptosis and increase the survival of neurons (45). Finally, physical exercise, besides affecting mitochondrial content, is also a strong stimulus for mitochondrial biogenesis (46). The current study indicated that T2DM increased ROS production in the diabetic brain mitochondria (Figure 3A). Also, Torabi et al. (47), Raza et al. (48), and Pintana et al. (49) reported increased ROS production in the mitochondria isolated from the brain in STZ-induced early T2DM rat model including a genetic model of T2DM, and a HFD-induced T2DM model, respectively. Increased ROS levels can cause mitochondrial dysfunction by damaging DNA and, thus, decreasing the expression of membrane proteins, especially mitochondrial electron transfer chain (ETC) complexes, and simultaneously increase in permeability of the mitochondria by opening the mitochondrial permeability transition pore (MPTP) (9, 11, 50-52). Conversely, the present study showed that physical exercise applied before the induction of diabetes significantly reduced mitochondrial ROS production in the diabetic rat's brain. These findings can be explained as follows exercise could increase the activity of complexes 1 and 3 in the ETC, leading to less ROS production (9, 16, 21). It is shown that exercise raises the mitochondrial antioxidant enzyme activity (44). In line with prior studies (47, 49), current results presented that MMP decreased in the brain of diabetic groups (Figure 3B). Mitochondrial ETC complexes cause MMP by pumping hydrogen ions into the inter-membrane space to continue ATP synthase and produce ATP with energy from this potential difference. Thus, the dysfunction of the ETC can lead to MMP collapse. Large amounts of ROS have been shown to trigger MPTP opening, which causes hydrogen ions to return to the matrix, leading to MMP collapse (50-53). Further, pre-exercise inhibited MMP collapse in the brain mitochondria of diabetic rats. A possible explanation for this result is that exercise improves mitochondrial ETC function, which may increase proton ion pumping into the inter-membrane space. Moreover, exercise may reduce MPTP opening by reducing ROS production, thereby preventing hydrogen ions from returning to the matrix (54).

The findings of our study showed more mitochondrial swelling in the diabetic groups, while exercise decreased mitochondrial swelling in these groups (Figure 3C). Brain mitochondrial swelling has also been found in T2DM induced by the high-fat diet (49). Swelling in the mitochondria occurs due to increased membrane permeability, damage to the membrane integrity due to lipid peroxidation, and opening of MPTP, all due to increased ROS levels in the mitochondria (50, 51). Therefore, exercise applied before the induction of diabetes may inhibit mitochondrial swelling by reducing ROS levels and membrane damage.

Also, this study indicated that brain mitochondrial outer membrane damage increased in the diabetic groups (Figure 3D). This increase in ROS production may cause oxidative stress and membrane lipid peroxidation, resulting in damage to the mitochondrial membrane (11). Further, our results revealed that exercise meaningfully reduced outer membrane damage in the brain. By reducing ROS production, exercise reduces membrane lipid peroxidation and decreases the severity of the membrane damage of mitochondria.

Our results showed that cytochrome c release significantly increased in diabetic groups, while exercise decreased cytochrome c release in these groups (Figure 3E). Increased cytochrome c release in diabetic rats may occur due to increased mitochondrial membrane permeability caused by increased ROS levels. Conversely, increased inhibition of ROS levels in diabetic rats who received exercise in the past may result in decreased mitochondrial membrane damage that causes reduced cytochrome c release.

**Figure 4.**
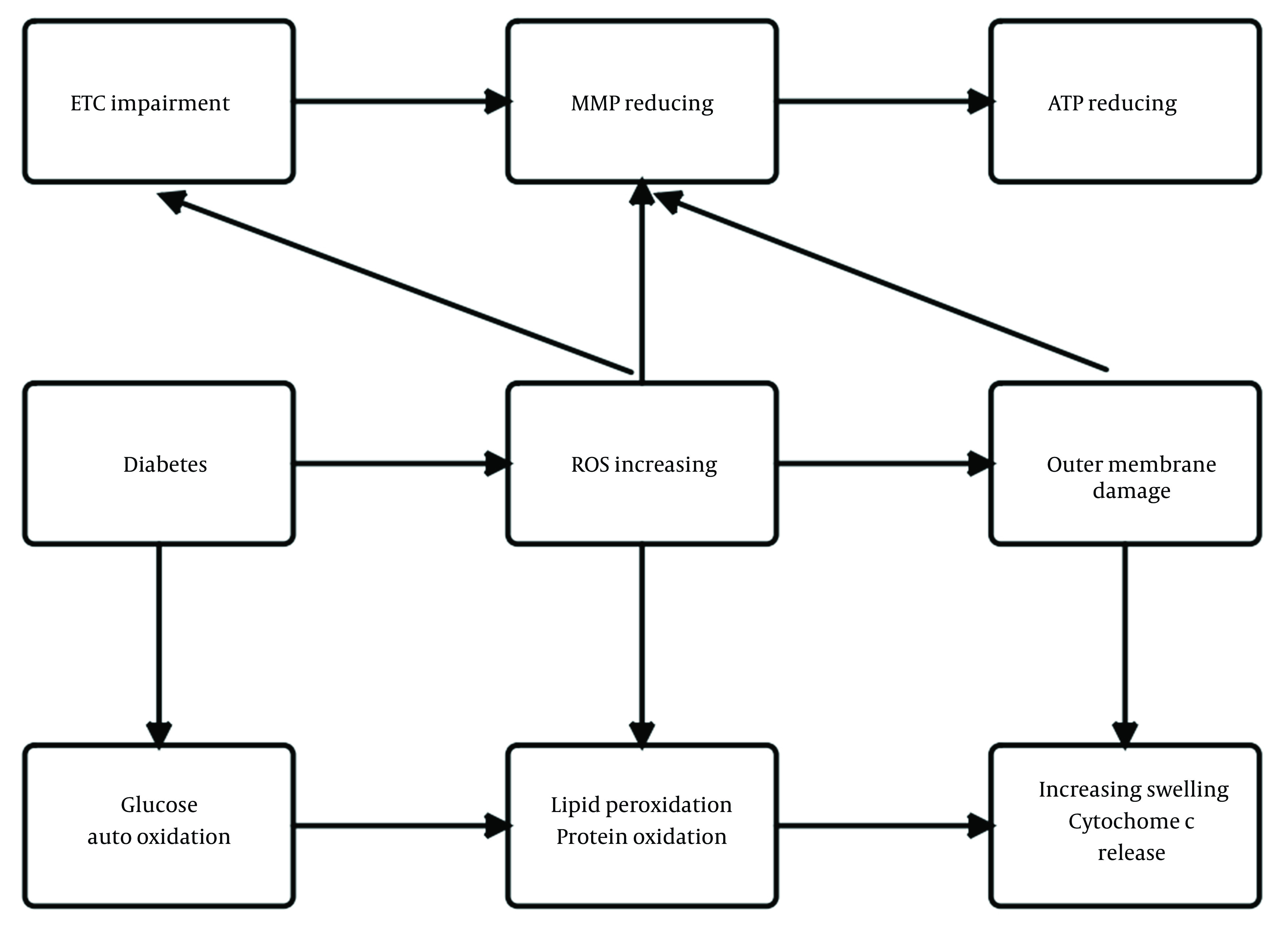
Relationship between mitochondrial parameters. ETC, electron transfer chain; MMP, mitochondrial membrane potential; ROS, reactive oxygen species

Lastly, this study’s results showed, ATP production decreased in the brain mitochondria of diabetic rats as evidenced by an increased ADP to ATP ratio (Figure 3F). Reduced ATP production has also been reported previously in the brain mitochondria of a genetic model T2DM (48). The brain is the largest energy user among the body's organs in proportion to weight. Continuous and abundant production of ATP is essential for proper brain function. Neuroplasticity required for learning and memory can be altered by changing the amount of produced or released ATP (23). The primary function of mitochondria is to produce ATP by oxidative phosphorylation through ETC. The decrease in MMP and increase in the permeability of the membrane of mitochondria with the simultaneous and destructive effect of ROS observed in the brain mitochondria of diabetic rats in the current study may result in decreased ATP production, which is seen as an increase in the ADP to ATP ratio. Our study also showed that exercise increased ATP production and, thus, reduced ADP to ATP ratio in the brain of diabetic rats, which can be explained by the increased efficiency of ATP production in mitochondrial ETC by improving the function of mitochondrial ETC enzymes due to exercise. Figure 4 shows a summary of the relationship between mitochondrial parameters measured in this study and diabetes.

### 5.1. Conclusions

The current study showed that T2DM causes spatial memory and learning impairment and dysfunction of the mitochondrial of the brain. Our study indicated the protective effects of physical exercise on spatial memory and learning impairments in T2DM rats. Our results also revealed physical exercise protective effects against dysfunction of the mitochondria of the brain in T2DM rats, which was demonstrated by inhibited MMP collapse, cytochrome c release, ROS production, outer membrane damage of the mitochondria, ADP to ATP ratio, and mitochondrial swelling in the brain of diabetic rats. Also, it should be noted that implications of tissue performance can be considered potentially important implications for tissue performance due to mitochondrial physiology. The authors considered that the effect of diabetes and physical exercise on different tissues and their mitochondria is dissimilar. Also, some mitochondrial functions (mitochondrial respiratory states; respiratory control ratio) were not assessed. Finally, our evidence supports that physical exercise before the onset of diabetes is promising in complications induced by diabetes.
